# An observational study on the association between smoking and relative poverty in China: Evidence from two waves of China Family Panel Studies

**DOI:** 10.18332/tid/210322

**Published:** 2025-11-04

**Authors:** Qiaoying Wei, Hao Wang, Quan Wan, Shenglin Liang, Wenpeng Pang, Qian Zeng, Peipei Chai

**Affiliations:** 1 Department of Post-graduation Medical Education, Guangxi Academy of Medical Sciences, Nanning, China; 2 Institute of Medical Information/Medical Library, Chinese Academy of Medical Sciences and Peking Union Medical College, Beijing, China; 3 China National Health Development Research Center, Beijing, China; 4 Department of Medical Education, The People's Hospital of Guangxi Zhuang Autonomous Region, Nanning, China; 5 Department of Microbiology, Basic Medical College, Guangxi Medical University, Nanning, China

**Keywords:** relative poverty, smoking household, Foster-Greer-Thorbecke Index, tobacco control

## Abstract

**INTRODUCTION:**

Smoking is the leading, preventable factor which significantly increases the likelihood of household relative poverty in China. This study aimed to explore the association between smoking and relative poverty across different households and provide evidence for targeted tobacco control measures and poverty reduction policies.

**METHODS:**

This study adopted a longitudinal design using two waves of unbalanced panel data from the China Family Panel Studies (CFPS) conducted in 2018 and 2020. Data were collected through structured questionnaires and self-reported responses. Smoking status of household members was considered the exposure factor, while household relative poverty status, measured by the Foster-Greer-Thorbecke indices, served as the outcome variable. A panel logit random effects model was employed to estimate the determinants of relative poverty across households with varying smoking status.

**RESULTS:**

At the 50% median income poverty line, China's relative poverty headcount ratio was 22.15% in 2018 and 22.54% in 2020, with the poverty gap index declining from 11.08% to 10.82% and the squared poverty gap index increasing slightly from 7.13% to 7.17%. Former-smoking households showed the highest poverty incidence (26.3% in 2018; 26.24% in 2020), followed by current-smoking (24.94%; 23.28%) and non-smoking households (22.75%; 22.37%). The panel logit model revealed significantly higher likelihood for current-smoking (adjusted odds ratio, AOR=1.63; 95% CI: 1.44–1.86, p<0.01) and former-smoking households (AOR=1.95; 95% CI: 1.60–2.36, p<0.01) compared to non-smoking households. Additional factors associated with increased odds of poverty included having ≥65 years members, members with chronic disease, and members reporting a two-week illness (all p<0.01).

**CONCLUSIONS:**

We conclude that China faces a substantial challenge of relative poverty, with tobacco use significantly increasing the likelihood of household poverty. Potential policy directions may include evaluating the effects of adjusting tobacco excise taxes and reforming tax collection mechanisms, exploring rural smokers’ preferences for smoking cessation information to inform the development of targeted interventions and so on.

## INTRODUCTION

Tobacco use remains the leading preventable cause of mortality worldwide^1^. China bears the highest global tobacco burden, with 26.6% of adults aged ≥15 years (over 300 million individuals) being current smokers – accounting for approximately one-third of the world’s smoking population^2,3^. As both the largest producer and consumer of tobacco products, China contributes 44% of global cigarette consumption, with per capita consumption 2.3 times the world average^4,5^. In China, smoking-attributable deaths surged by 57.9%, from 1.5 million in 1990 to 2.4 million in 2019^6^.

The health consequences of tobacco use are severe and dose-dependent, elevating risks for cardiovascular, respiratory, and other diseases with prolonged exposure^7^. Epidemiological studies reveal alarming trends: age-standardized disability-adjusted life years among Chinese males reached 6424.83 per 100000 in 2017 – a 41.25% increase since 1990^8^. Beyond health impacts, tobacco use erodes human capital by impairing labor productivity, ultimately reducing individual earnings and exacerbating income poverty^9^. The World Health Organization identifies three poverty pathways: 1) diversion of household expenditure from essentials to tobacco, 2) catastrophic healthcare costs for tobacco-related illnesses, and 3) income loss from premature mortality^10^. Empirical evidence confirms these mechanisms in China: current and former smokers face income reductions of 37.70% and 44.00%, respectively^11^, while cigarette expenditures elevate urban and rural poverty rates by 6.4% and 1.9%^12^. Smoking households also demonstrate significantly higher risks of catastrophic health spending^13^. Collectively, these findings underscore tobacco’s dual threat – undermining population health while exacerbating socioeconomic inequalities.

Existing research predominantly examines tobacco’s role in absolute poverty, a challenge China officially resolved in 2020. However, relative poverty persists as an enduring policy concern^14^. This study advances the literature by employing the Foster-Greer-Thorbecke (FGT) index to analyze smoking’s impact on relative poverty dynamics^15^. Our findings aim to inform targeted tobacco control strategies and evidence-based anti-poverty policies in the post-elimination era, aligning with Healthy China 2030 objectives.

## METHODS

### Data source

The study data are derived from the 2018 and 2020 waves of the China Family Panel Studies (CFPS), a nationally representative longitudinal survey. All study variables, including households’ socioeconomic status, demographic characteristics, health conditions, and smoking behavior, were based on self-reported information obtained via questionnaires. By matching households’ identifiers across two waves, we construct an unbalanced panel database. The CFPS questionnaire provides comprehensive measures requisite for computing the Foster-Greer-Thorbecke (FGT) indices. With a sampling frame encompassing 25 provincial-level administrative units (including provinces, autonomous regions, and direct-administered municipalities) and over 900 county-level districts, the CFPS offers extensive geographical coverage, ensuring reliable representativeness of China’s diverse socioeconomic landscape.

### Variables

*Definitions*

Households are divided into three mutually exclusive categories based on smoking status: 1) Current-smoking households – at least one member is a current smoker, defined as an individual who has smoked ≥100 cigarettes lifetime and consumed ≥1 cigarette daily in the 30 days preceding the survey; 2) Non-smoking households – no members meet the criteria for current or former smokers; and 3) Former-smoking households – all other cases (i.e. households with former smokers but no current smokers).

The analysis focused on households that met the following inclusion criteria: 1) surveyed in the 2018 and/or 2020 CFPS waves with valid households’ identifiers, allowing proper panel construction; and 2) provided complete information on household’s poverty status and key covariates, including smoking status, demographic characteristics, and health-related variables.

Households were excluded if they met any of the following criteria: 1) missing or inconsistent identifiers across waves, preventing reliable matching; 2) incomplete or missing data on smoking status or poverty indicators; and 3) reporting implausible or extreme values for economic or demographic variables (e.g. negative income or unrealistic household size).

*Household income*

This study uses household income to measure the Foster-Greer-Thorbecke(FGT) indices^15^. Household income in the CFPS is aggregated from five constituent sources:

- Labor income: post-tax wages, bonuses, and in-kind benefits from agricultural/non-agricultural employment.
- Operational income: net profits from agricultural/forestry/livestock/aquatic production (after cost deduction), home-produced-and-consumed goods, and family-run businesses/private enterprises.
- Transfer income: government transfers (e.g. pensions, subsidies, welfare) and social assistance.
- Property income: earnings from leasing land, housing, or productive assets.
- Other income: private transfers (e.g. gifts, remittances) and miscellaneous receipts.

*Foster-Greer-Thorbecke index (FGT)*

Relative poverty is measured by the Foster-Greer-Thorbecke (FGT) index, which is multidimensional and incorporates the three indices of Headcount Ratio, Poverty Gap Index, and Squared Poverty Gap Index^16^. Specifically, households are ranked in ascending order of income, and the poverty line is defined as 50% of the median household income. Households with an income below this threshold are classified as relatively poor and assigned a value of 1, whereas those at or above the threshold are assigned a value of 0. FGT is an international poverty measure that has been widely used in the empirical poverty literature because of its simple additive structure, which allows for decomposing the index and analyzing each subgroup individually. The index is constructed based on normalized income shortfalls, with its general formula expressed as:


$$\text{FGT}=\frac{1}{\text{n}}{\Sigma_{\text{i=1}}^{\text{q}}\left(\frac{\text{z}-{\text{y}}_{\text{i}}}{\text{Z}}\right)}^{\text{a}}$$

where, *y*_i_ is income of household *i*, *z* is relative poverty line (income threshold), *n* is total number of households, *q* is number of households in poverty (where *y*_i_<*z*), and *α* is poverty aversion parameter (higher values place greater weight on the most severely impoverished households).

- FGT0 (α=0): Headcount Ratio (q/n), measures the incidence of poverty – the proportion of households below the poverty line.
- FGT1 (α=1): Poverty Gap Index, captures the depth of poverty, quantifying the average shortfall of poor households’ incomes relative to z.
- FGT2 (α=2): Squared Poverty Gap Index, reflects the severity of poverty, emphasizing inequality among the poor by squaring normalized gaps.

*Relative poverty line specification*

While there is no universal relative poverty standard, international practice (e.g. in the European Union and Organization for Economic Co-operation and Development (OECD) countries such as Japan, Australia, and the United Kingdom (UK) commonly adopts thresholds of 50% or 60% of median household income^17,18^. Aligning with prior studies on China, the study defines the poverty line as 50% of median household income^14^. To ensure reliability, the study also estimate poverty indices using a stricter threshold (2/3 of median income).

### Empirical model

Given that the dependent variable, household relative poverty, is a binary indicator, we employ a binary logit model to estimate its determinants. Following the Hausman test, we adopt panel logit random effects model to account for unobserved household heterogeneity and the problem of endogeneity. The model specification is as follows:


$$\text{Logit}\left(Y_{it}\right)=\beta_{0}+\beta_{i}\left(Smoking_{it}\right)+\beta_{2}X_{it}+a_{i}+u_{i}t$$

where *Y*_it_ is binary poverty status of household *i* in year *t* (1 if impoverished, 0 otherwise). *Smoking*_it_ is assigned as follows: 0 for non-smoking households (reference group), 1 for current-smoking households, and 2 for former-smoking households. *X*_it_ is a vector of control variables, including residence (rural, non-rural), having elderly people aged ≥65 years in household (yes, no), having household members with chronic disease (yes, no), having alcoholic members in household (yes, no), having hospitalized members in household (yes, no), having medical insurance covered members in household (yes, no), having two-week illness members in household (yes, no), household size (1–2, 3–4, ≥ 5 persons), location of China (East, Central, West, Northeast), having employee members in household (0, 1, 2, ≥ 3 persons).

### Statistical analysis

Data cleaning and analysis were performed using STATA 18.0. Descriptive statistics for categorical variables (e.g. residence, chronic disease prevalence, recent illness), reported as frequencies and percentages. A panel logit random effects model was employed to estimate the determinants of relative poverty across households with varying smoking status. The significance level was p<0.05.

## RESULTS

### Demographic characteristics

The study included 13456 households in 2018 and 11036 households in 2020. In both years, around 44% of households were non-smoking, with current-smoking households decreasing from 50.04% in 2018 to 44.49% in 2020, and former-smoking households increasing from 5.71% to 11.36%. Rural households accounted for 59.69% in 2018 and 55.74% in 2020. About one-third of households had members aged ≥65 years, and roughly one-quarter had members with chronic diseases in both waves. Households with hospitalized members decreased from 24.49% to 17.24% and those reporting illness in the past two weeks decreased from 49.13% to 38.93%. Geographically, households were distributed across eastern, central, western, and northeastern China, with minimal changes between waves. On average, households had approximately 1.9 employed members in 2018 and 1.8 in 2020 (Table 1).

**Table 1:** Descriptive statistics of study variables, an observational study conducted in China from 2018 (N=13456) to 2020 (N=11036)

*Variables*	*2018 (N=13456)n (%)*	*2020 (N=11036)n (%)*
**Household smoking category**		
Non-smoking	5955 (44.25)	4872 (44.15)
Current-smoking	6733 (50.04)	4910 (44.49)
Former-smoking	768 (5.71)	1254 (11.36)
**Residence**		
Rural	8032 (59.69)	6151 (55.74)
Non-rural	5424 (40.31)	4885 (44.26)
**Having elderly people aged** ≥**65 years in household**		
No	8687 (64.56)	7037 (63.76)
Yes	4769 (35.44)	3999 (36.24)
**Household members with chronic disease**		
No	9987 (74.22)	8383 (75.96)
Yes	3469 (25.78)	2653 (24.04)
**Alcoholic members in household**		
No	9693 (72.03)	8604 (77.96)
Yes	3763 (27.97)	2432 (22.04)
**Having hospitalized members in household**		
No	10160 (75.51)	9133 (82.76)
Yes	3296 (24.49)	1903 (17.24)
**Having medical insurance covered members in household**		
No	1321 (9.82)	1400 (12.69)
Yes	12135 (90.18)	9636 (87.31)
**Having two-week illness members in household**		
No	6845 (50.87)	6740 (61.07)
Yes	6611 (49.13)	4296 (38.93)
**Household size** (number of persons)		
1–2	4359 (32.39)	3539 (32.07)
3–4	5130 (38.13)	4191 (37.98)
≥5	3967 (29.48)	3306 (29.96)
**Location of China**		
East	4711 (35.01)	3955 (35.84)
Central	3128 (23.25)	2560 (23.20)
West	3768 (28.00)	3020 (27.36)
Northeast	1849 (13.74)	1501 (13.60)
**Having employee members in household**		
0	1721 (12.79)	1693 (15.34)
1	3907 (29.04)	3612 (32.73)
2	3840 (28.54)	2849 (25.82)
≥3	3988 (29.64)	2882 (26.11)

### FGT indices distribution under the 50% median income poverty line

The relative poverty headcount ratio among Chinese households remained stable at 22.15% (2018) and 22.54% (2020), while the poverty gap index showed marginal improvement (11.08% to 10.82%) and the squared poverty gap index slightly increased (7.13% to 7.17%). Disaggregated analysis revealed significant disparities across household types: in 2018, former-smoking (26.3%) and current-smoking households (24.94%) showed 3.55 and 2.18 percentage-point

higher poverty rates, respectively, than non-smoking households. By 2020, former-smoking households maintained the highest poverty incidence (26.24%), while the gap between current-smoking (23.28%) and non-smoking households (22.37%) narrowed. Specifically, former-smoking households consistently demonstrated the deepest poverty (poverty gap index: 12.92% in 2018; 12.34% in 2020), followed by current-smoking (11.28%; 10.13%) and non-smoking households (10.88%; 11.26%). All three households’ types showed comparable poverty intensity, with squared poverty gap indices fluctuating around 7% during both periods (Table 2).

**Table 2:** FGT indices distribution under the 50% median income poverty line (%), an observational study conducted in China from 2018 (N=13456) to 2020 (N=11036)

*Household smoking category*	*2018*				*2020*			
	*n*	*FGT0*	*FGT1*	*FGT2*	*n*	*FGT0*	*FGT1*	*FGT2*
Current-smoking	6733	24.94	11.28	7.09	4910	23.28	10.13	6.18
Former-smoking	768	26.30	12.92	8.88	1254	26.24	12.34	8.17
Non-smoking	5955	22.75	10.88	7.02	4872	22.37	11.26	7.93
Total	13456	22.15	11.08	7.13	11036	22.54	10.82	7.17

FGT0, FGT1, and FGT2 represent the headcount ratio, poverty gap index, and squared poverty gap index, respectively.

Analysis by household residence reveals divergent trends: rural households showed a decline in poverty incidence (FGT0) from 24.63% (2018) to 20.84% (2020), while urban households experienced an increase from 21.83% to 22.99%. Significant disparities emerged when examining smoking status: in 2018, rural current-smoking (24.76%) and former-smoking households (25.42%) showed higher poverty rates than their urban counterparts (22.33% and 24.81%, respectively). Non-smoking households demonstrated lower poverty rates in both groups (rural: 24.61%; urban: 21.7%). By 2020, rural former-smoking households saw their poverty rate rise to 26.64%, whereas urban former-smoking households improved to 22.49%. Non-smoking households maintained relatively stable poverty indices, with rural and urban rates at 21.99% and 21.47%, respectively, in 2020 (Table 3).

**Table 3:** FGT indices distribution by residence under the 50% median income poverty line (%), an observational study conducted in China from 2018 (N=13456) to 2020 (N=11036)

*Household smoking category*	*Residence*	*2018*				*2020*			
		*n*	*FGT0*	*FGT1*	*FGT2*	*n*	*FGT0*	*FGT1*	*FGT2*
**Current-smoking**	Rural	4431	24.76	12.23	7.76	3057	22.96	9.99	6.24
	Non-rural	2302	22.33	8.77	5.10	1853	21.96	9.18	5.28
**Former-smoking**	Rural	236	25.42	12.65	8.24	747	26.64	14.10	9.37
	Non-rural	532	24.81	13.27	9.31	507	22.49	8.59	4.97
**Non-smoking**	Rural	3365	24.61	12.02	7.83	2347	21.99	12.40	8.65
	Non-rural	2590	21.70	9.38	5.75	2525	21.47	10.66	7.49
**Total of rural**		8032	24.63	11.49	7.35	6151	20.84	11.11	7.43
**Total of non-rural**		5424	21.83	9.45	5.81	4885	22.99	9.84	6.34

FGT0, FGT1, and FGT2 represent the headcount ratio, poverty gap index, and squared poverty gap index, respectively.

### FGT indices distribution under the 2/3 median income poverty line

When applying the more stringent poverty threshold (2/3 of median income), China’s relative poverty headcount ratio (FGT0) showed a marginal increase from 31.82% in 2018 to 32.4% in 2020, while poverty depth (FGT1) and severity (FGT2) demonstrated modest improvements, declining from 15.46% to 15.17% and 9.88% to 9.75%, respectively. Disaggregated analysis revealed persistent disparities by smoking status: both former-smoking (34.25%) and current-smoking households (34.93%) showed higher poverty incidence than non-smoking households (32.9%) in 2018, with gaps of 1.93 and 2.02 percentage points. By 2020, this pattern remained largely unchanged, though the differential between smoking and non-smoking households (32.41%) narrowed slightly. It is observed that former-smoking households consistently displayed the greatest poverty depth (FGT1: 17.12% in 2018; 16.74% in 2020) and severity (FGT2: 11.62%; 11%), while current-smoking households showed intermediate values, and non-smoking households the most stable indicators across all three dimensions (Table 4).

**Table 4:** FGT indices distribution under the 2/3 median income poverty line (%), an observational study conducted in China from 2018 (N=13456) to 2020 (N=11036)

*Household smoking category*	*2018*			*2020*		
	*FGT0*	*FGT1*	*FGT2*	*FGT0*	*FGT1*	*FGT2*
Current-smoking	34.16	15.71	9.93	34.24	14.84	8.99
Former-smoking	34.25	17.12	11.62	34.93	16.74	11.00
Non-smoking	32.23	15.15	9.67	32.41	15.21	10.26
Total	31.82	15.46	9.88	32.40	15.17	9.75

FGT0, FGT1, and FGT2 represent the headcount ratio, poverty gap index, and squared poverty gap index, respectively; the same sample size was used under both the 2/3 and 50% poverty line criteria.

In 2018 and 2020, the relative poverty incidence (FGT0) among Chinese households was 31.82% and 32.4%, respectively. The poverty gap (FGT1) declined from 15.46% to 15.17%, while the squared poverty gap (FGT2) decreased from 9.88% to 9.75%. Analysis by household smoking status reveals significant differences. In 2018, both former-smoking households (34.25%) and current-smoking households (34.93%) showed higher poverty rates than non-smoking households (32.41%), exceeding the latter by 1.93 percentage points and 2.02 percentage points, respectively. By 2020, the poverty rates for current-smoking and former-smoking households were 34.24% and 34.93%, respectively, showing a smaller gap between them. Non-smoking households maintained the lowest poverty rate at 32.41%. Over the two-year period, former-smoking households consistently recorded the highest poverty gap (FGT1), at 17.12% (2018) and 16.74% (2020). The FGT1 for current-smoking households was 15.71% (2018) and 14.84% (2020), while non-smoking households registered 15.15% (2018) and 15.21% (2020). Similarly, former-smoking households had the highest squared poverty gap (FGT2) in both years (11.62% in 2018 and 11% in 2020). The FGT2 for both current-smoking households and non-smoking households fluctuated around 9% (Table 5).

**Table 5:** FGT indices distribution by residence under the 2/3 median income poverty line (%), an observational study conducted in China from 2018 (N=13456) to 2020 (N=11036)

*Household smoking category*	*Residence*	*2018*			*2020*		
		*FGT0*	*FGT1*	*FGT2*	*FGT0*	*FGT1*	*FGT2*
**Current-smoking**	Rural	33.90	16.75	10.76	32.54	13.85	8.06
	Non-rural	32.19	13.42	7.76	33.30	14.49	8.89
**Former-smoking**	Rural	36.44	17.26	11.20	32.74	13.16	7.58
	Non-rural	34.40	17.49	12.01	36.15	18.46	12.42
**Non-smoking**	Rural	34.98	16.36	10.64	30.42	14.78	9.82
	Non-rural	32.01	13.93	8.36	32.47	16.49	11.23
**Total of rural**		33.21	15.75	10.12	32.24	14.26	8.87
**Total of non-rural**		31.14	13.90	8.42	31.30	15.28	10.01

FGT0, FGT1, and FGT2 represent the headcount ratio, poverty gap index, and squared poverty gap index, respectively; the same sample size was used under both the 2/3 and 50% poverty line criteria.

### Random effects model of relative poverty headcount ratio

The panel logit random effects model revealed significant associations between household characteristics and relative poverty likelihood. Compared to non-smoking households, both current-smoking (adjusted odds ratio, AOR=1.63; 95% CI: 1.44–1.86, p<0.01) and former-smoking households (AOR=1.95; 95% CI: 1.60–2.36, p<0.01) showed substantially higher odds of experiencing relative poverty. Several likelihoods factors emerged as statistically significant predictors: presence of elderly members aged ≥65 years (AOR=3.25; 95% CI: 2.86–3.70, p<0.01), chronic disease patients in household (AOR=1.44; 95% CI: 1.27–1.63, p<0.01), and recent illness episodes within two weeks (AOR=1.37; 95% CI: 1.22–1.54, p<0.01) (Table 6).

**Table 6:** Panel logit random effects model results of association between smoking and relative poverty, an observational study conducted in China from 2018 (N=13456) to 2020 (N=11036)

*Variables*	*AOR*	*SE*	*z*	*p*	*95% CI*	
					*Lower*	*Upper*
**Household smoking category** (reference: Non-smoking)						
Current-smoking	1.63	0.11	7.46	<0.01	1.44	1.86
Former-smoking	1.95	0.19	6.74	<0.01	1.60	2.36
**Residence** (reference: Rural)						
Non-rural	0.23	0.01	-22.49	<0.01	0.20	0.26
**Household person aged** ≥**65 years** (reference: No)						
Yes	3.25	0.21	17.98	<0.01	2.86	3.70
**Household members with chronic disease** (reference: No)						
Yes	1.44	0.09	5.70	<0.01	1.27	1.63
**Alcoholic household members** (reference: No)						
Yes	0.81	0.06	-3.13	0.00	0.70	0.92
**Inpatient service** (reference: No)						
Yes	1.00	0.07	0.04	0.964	0.88	1.15
**Medical insurance covered members** (reference: No)						
Yes	0.77	0.07	-2.86	0.004	0.64	0.92
**Employee members** (reference: 0 persons)						
1	0.39	0.03	-10.53	<0.01	0.33	0.46
2	0.29	0.03	-12.38	<0.01	0.24	0.35
≥3	0.16	0.02	-15.97	<0.01	0.12	0.20
**Two-week illness** (reference: No)						
Yes	1.37	0.08	5.38	<0.01	1.22	1.54
**Year** (reference: 2018)						
2020	0.58	0.03	-10.89	<0.01	0.53	0.64
**Family size** (reference :1–2 persons)						
3–4	0.26	0.02	-17.99	<0.01	0.23	0.31
≥5	0.11	0.01	-23.28	<0.01	0.09	0.14
**Location** (reference: East)						
Central	2.43	0.21	10.44	<0.01	2.06	2.87
West	3.97	0.33	16.54	<0.01	3.37	4.68
Northeast	1.89	0.18	6.48	<0.01	1.56	2.29
Constant	0.47	0.06	-6.42	<0.01	0.38	0.59

AOR: adjusted odds ratio. SE: standard error.

## DISCUSSION

This study employed data from the China Family Panel Studies (CFPS) to estimate poverty indices for households with different smoking statuses under two poverty thresholds (50% and 2/3 of median household income). Using a panel logit random effects model, we examined the association between households smoking behavior and relative poverty. These findings provide evidence for targeted tobacco control measures and poverty reduction policies in China.

The results demonstrate that compared to non-smoking households, both current-smoking and former-smoking households have significantly higher likelihood of falling into relative poverty. This phenomenon may occur because households with tobacco expenditures sacrifice basic needs by diverting limited resources from healthcare, education, and food to support addictive smoking behaviors, thereby exacerbating poverty likelihood. As estimated by John et al.^19^, approximately 15 million people in India were pushed into poverty due to tobacco-related expenditures. Furthermore, smoking-induced health problems may lead to increased medical expenses and reduced work capacity, resulting in household economic instability and decreased income^20^.

In absolute terms of poverty indices, rural current-smoking and former-smoking households showed higher relative poverty rates in 2020 compared to their urban counterparts. This disparity may stem from higher smoking prevalence in rural households and greater proportion of households expenditures allocated to tobacco, magnifying tobacco’s poverty impact in rural areas^21^. With lower baseline incomes than urban households, rural families face higher opportunity costs from smoking, and after tobacco expenditures their remaining income often falls below the poverty line^22^. Additionally, rural residents bear the heaviest tobacco-related burdens, with higher susceptibility to smoking-related diseases and consequent healthcare costs, exposing their households to greater risks of both transient and chronic poverty^23^.

Across households’ types, former-smoking households consistently showed the highest relative poverty indices under both poverty thresholds during the two-year period. This suggests that while smoking cessation benefits health, the long-term economic consequences (e.g. accumulated medical debt, prior resource misallocation) may persistently affect household economic status. The logit regression results confirm that former-smoking households face higher relative poverty likelihood. This may relate to smokers’ status transition f as they become aware of smoking’s severe health consequences, their cessation attitudes intensify, increasing likelihood of transitioning from current to Former-smoking status. Wang et al.^24^ found that 68.79% of smokers developed cessation intentions following doctors’ advice, while 77.78% considered quitting after developing at least one chronic disease.

### Recommendations

Based on these findings, the following recommendations are suggested for future directions to reduce the smoking burden. First, enhancing tax transparency and improving collection mechanisms could be considered, drawing on international experience while taking into account China’s national context. China’s current cigarette tax rate of 56% of retail price remains below WHO’s recommended 75% threshold^4^. Studies show that a 10% reduction in tobacco affordability could decrease consumption by 1.65%, prevent numerous premature deaths, increase tax revenue, and reduce poverty incidence^25^. Currently, China implements hidden tobacco taxes where levies are embedded in product prices, creating opaque tax burdens^26^. In contrast, countries like the US and Japan employ price-tax separation, allowing consumers to clearly identify excise taxes^27^. Moreover, while China primarily uses ad valorem taxation, most OECD countries rely predominantly on specific excise taxes^27^. Brazil and France employ a hybrid system with specific excise taxes as the main component^28^. The US implements dual taxation with federally mandated uniform excises plus additional local specific taxes^29^.

Second, focus on rural smokers by investigating their preferences for cessation messages and developing visual anti-smoking communications to promote voluntary quitting. China’s cigarette packaging warnings have remained unchanged for years, likely inducing message fatigue and diminishing health warnings’ effectiveness. Overfamiliar anti-tobacco message frames may activate greater message fatigue and resistance to persuasion, which may dampen campaign effects^30^. International experience shows that warning labels with images that elicit more negative emotional reaction are associated with increased risk perceptions and quit intentions in adults relative to text-only warnings^31^. However, graphic warnings containing images which evoke little emotional reaction can backfire and reduce risk perceptions and quit intentions versus text-only warnings^31^.

Third, continued efforts in tobacco control education and the exploration of national-level anti-smoking legislation may play a role in reducing smoking prevalence. The ‘Healthy China 2030’ blueprint explicitly targets 80% population coverage by comprehensive smoke-free laws by 2030^32^. We recommend enacting nationwide smoke-free legislation. China’s current tobacco control laws are primarily regional, with relatively low legal hierarchy and slow legislative progress. Currently, over 20 cities have taken promising steps to enact laws complying with WHO FCTC requirements^33^. However, smoke-free laws of some cities still fail to meet FCTC standards, requiring strengthened content and enforcement. Shanghai’s comprehensive smoking ban experience suggests that if a nationwide public smoking ban was implemented in China between 2017 and 2035, economic gains of RMB 1675.98 billion (approximately US$248.3 billion) would be observed^34^. Therefore, promulgating national smoke-free legislation appears to be an important direction for strengthening tobacco control.

### Limitations

This study has several limitations that should be acknowledged. First, our measure of relative poverty relies on 50% and two-thirds of median household income, which does not fully capture multidimensional aspects such as education, health, housing, or social security, and may underestimate the broader impact of smoking. Second, key variables – including household income, smoking status, and health conditions – were self-reported, which could lead to recall bias and misclassification of both exposure and outcome. Third, despite adjusting for observable confounders, residual confounding may remain, and reverse causality cannot be ruled out. Finally, although CFPS is nationally representative for China, the findings may have limited generalizability to other countries or specific subpopulations.

## CONCLUSIONS

This study examines smoking’s impact on relative poverty using CFPS 2018–2020 data. The panel logit random effects model shows current-smoking and former-smoking households face significantly higher relative poverty likelihood than non-smoking households. This indicates smoking constitutes not only a major health threat but also a key socioeconomic factor exacerbating household economic vulnerability and relative poverty likelihood. Therefore, reducing smoking prevalence, particularly by protecting low-income populations from tobacco harm, may serve as a potential strategy for alleviating household economic burdens and addressing intergenerational poverty. However, further research is needed to provide sufficient evidence on its effectiveness in different contexts.

## Data Availability

The data supporting this research are available from the authors on reasonable request.
